# Carbon System Measurements and Potential Climatic Drivers at a Site of Rapidly Declining Ocean pH

**DOI:** 10.1371/journal.pone.0053396

**Published:** 2012-12-28

**Authors:** J. Timothy Wootton, Catherine A. Pfister

**Affiliations:** Department of Ecology and Evolution, The University of Chicago, Chicago, Illinois, United States of America; University of California, Merced, United States of America

## Abstract

We explored changes in ocean pH in coastal Washington state, USA, by extending a decadal-scale pH data series, by reporting independent measures of dissolved inorganic carbon (DIC), spectrophotometric pH, and total alkalinity (TA), by exploring pH patterns over larger spatial scales, and by probing for long-term trends in environmental variables reflecting potentially important drivers of pH. We found that pH continued to decline in this area at a rapid rate, that pH exhibited high natural variability within years, that our measurements of pH corresponded well to spectrophotometric pH measures and expected pH calculated from DIC/TA, and that TA estimates based on salinity predicted well actual alkalinity. Multiple datasets reflecting upwelling, including water temperature, nutrient levels, phytoplankton abundance, the NOAA upwelling index, and data on local wind patterns showed no consistent trends over the period of our study. Multiple datasets reflecting precipitation change and freshwater runoff, including precipitation records, local and regional river discharge, salinity, nitrate and sulfate in rainwater, and dissolved organic carbon (DOC) in rivers also showed no consistent trends over time. Dissolved oxygen did not decline over time, indicating that long-term changes did not result from shifts in contributions of respiration to pH levels. These tests of multiple potential drivers of the observed rapid rate of pH decline indicate a primary role for inorganic carbon and suggest that geochemical models of coastal ocean carbon fluxes need increased investigation.

## Introduction

There has been strong recent interest in the carbon cycle of marine environments, because of the large role oceans play in the carbon cycle. The consequences of the substantial oceanic uptake of anthropogenic releases of CO_2_ into the atmosphere has been of particular recent interest, because the introduction of CO_2_ into the oceans changes ocean chemistry, including lowering pH, which may affect key biological processes such as skeletal and reef calcification. There is accumulating direct evidence that ocean pH has declined over the past two decades [1]–[5], and that reduced pH can affect selected species in the lab [6]–[8], key species in their natural environment [9] and the structure and function of marine ecosystems [2], [9], [10].

Global models based on physical models of the ocean, historic measurements and projections of atmospheric CO_2_, and chemical equilibria of inorganic components of the ocean carbon system predict that ocean pH should decline at a rate of −0.002 to −0.004 units per year, with an acceleration over time as CO_2_ release increases and buffering capacity of the oceans becomes depleted [11]–[16]. Observed rates from low-latitude, offshore sites (Bermuda [4], Canary Islands [5], Hawaii [3]) show similar rates of decline over the past two decades. In contrast, time series from several high-latitude coastal areas (Washington state, USA [2], Netherlands [17]) exhibit much faster rates of pH decline than predicted in recent decades. In light of the important roles these temperate coastal systems play in ocean and fisheries production, probing the causes of these declines is critical. Although explanations have been asserted for pH declines that are greater than expected, there have been no tests of possible environmental drivers by following their changes through time. For example, Feely et al. [18] report low pH and high pCO_2_ concentrations in the Strait of Juan de Fuca, which corroborates the data of Wootton et al. [2], and conclude on the basis of spatially distributed patterns of pH with depth [19] that altered upwelling has increased CO_2_ delivery to the region. While such space-for-time substitutions can be powerful for identifying potential mechanisms of change [20], the evidence they provide is not as strong as direct observations of temporal change in potential drivers, because the former leaves open the possibility that alternative drivers cause the trend. Similarly, after also reporting rapid pH decline in the North Sea, Provoost et al. [17] conclude that increased eutrophication, rather than changing upwelling, is the driver. While changes in currents, upwelling, or nutrient loading seem equally plausible, temporal data were not presented in either case. Therefore, more intensive analyses of temporal changes in putative drivers of ocean pH other than CO_2_ are critical to better understand why pH appears to be declining rapidly in high-latitude coastal locales.

In this paper we temporally and spatially extend the pH data series from the northeastern Pacific off the coast of Washington state [2]. We supplement these data with independent data on spectrophotometric pH, pCO_2_ and total alkalinity (TA) to more fully describe the dissolved inorganic carbon (DIC) system at this site. Finally, we probe multiple data series for long-term trends that test several alternative hypotheses to explain rapid pH decline at this site. These hypotheses include:

1. *Upwelling has increased*, bringing water high in DIC derived from respiration [19], [20]. This hypothesis predicts increasing values of the coastal upwelling index, increasing nitrate and phosphorus concentrations, declining water temperatures, declining dissolved oxygen (DO) concentrations, and gradual changes in local surface winds over time.
2. *Eutrophication has increased,* presumably as a result of increasing anthropogenic nutrients, causing increased CO_2_ content as the products of primary production decay [17]. This hypothesis also predicts increasing nutrient concentrations, increasing algal blooms, and declining oxygen concentrations over time.
3. *Climate change increases freshwater input*, which dilutes the buffering capacity of the ocean [21]. This hypothesis predicts declining salinity, increasing rainfall, and increasing river discharge over time.
4. *Increasing input of dissolved organic carbon (DOC) from land* [*22*] *increases oceanic respiration, lowering pH.* This hypothesis predicts declining dissolved oxygen and increasing DOC in rivers in the region.
5. *Low pH arises from input of acids other than CO_2_-derived carbonic acid*, such as acid precipitation from increased emissions or changes in input of organic acids from biological processes. This hypothesis predicts declining total alkalinity. In the case of acid precipitation input, the hypothesis predicts long-term increases in sulfate and nitrate concentrations in precipitation.
6. *Changes in phytoplankton abundance change pH.* Decreasing phytoplankton might reduce organic carbon fixation, reducing inorganic carbon uptake and reducing pH. Phytoplankton has been suggested to be in global decline [23]. Alternatively, an increasing magnitude of algal blooms might increase transport of organic carbon to sub-surface zones, where it could decompose and decrease the pH of upwelled surface water.

## Results

### Carbon System Measurements

**Figure 1:**
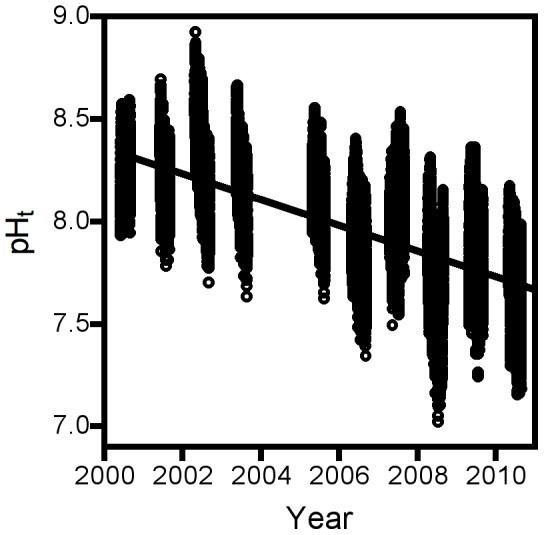
Trends in ocean pH taken from Tatoosh Island, Washington, from 2000–2010, expressed on the total scale. 2004 not reported because of probe failure. N = 37,038.

**Figure 2:**
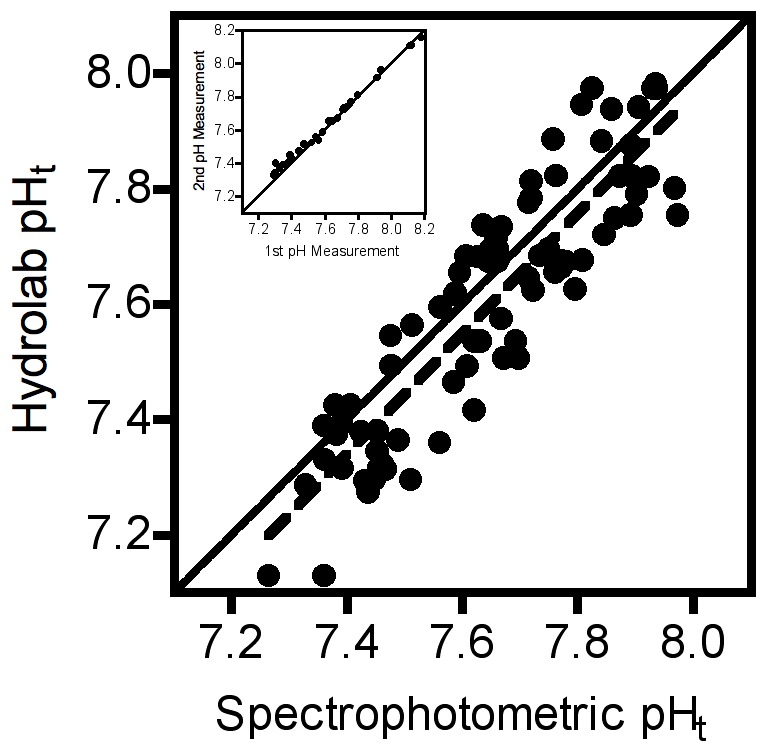
Relationship between pH of water samples measured using spectrophotometric methods and pH measured in situ with an electrochemical probe at the time of water collection. Inset: repeatability of spectrophometric pH measurements. Solid lines: identity relationship. Dashed lines: lines of best fit from linear regression.

Sampled pH continued to decline at Tatoosh Island (Fig. 1) at a rate of −0.058 units per year. Our spectrophotometric measures of pH proved to be highly repeatable (r = 0.997, Fig. 2 inset), but close inspection revealed a slight systematic deviation from the identity relationship, yielding the equation (pH_2nd_ = 0.62+0.92 pH_1st_, where pH_1st_ and pH_2nd_ correspond to the first and second measurements, respectively; linear regression, p(pH_2nd_ = pH_1st_) <0.0001). Hence at low pH values, subsequent measurements tended to be slightly higher than expected whereas at high pH values, subsequent measurements tended to be lower than expected. Second measurements were made after all first measurements were made; hence, samples could interact for approximately 15–30 min with an air headspace introduced after the first measurement was made. We therefore interpret this deviation as the result of DIC in the water starting to equilibrate with CO_2_ in the air. Overall, pH measured by the electrochemical probe corresponded to pH measured spectrophotometrically in the lab (r = 0.903, Fig. 2), and did not deviate significantly from the identity relationship (linear regression, pH_HL_ = −0.202+1.019 pH_spec_, p(pH_HL_ = pH_spec_) = 0.709), indicating that our in-situ electrochemical measurements provided reasonable and unbiased pH estimates, albeit with the larger error for each measurement expected by the method. The pH probe also measured seawater buffer with good accuracy (measured: 8.103±0.012, observed: 8.108±0.001).

**Figure 3:**
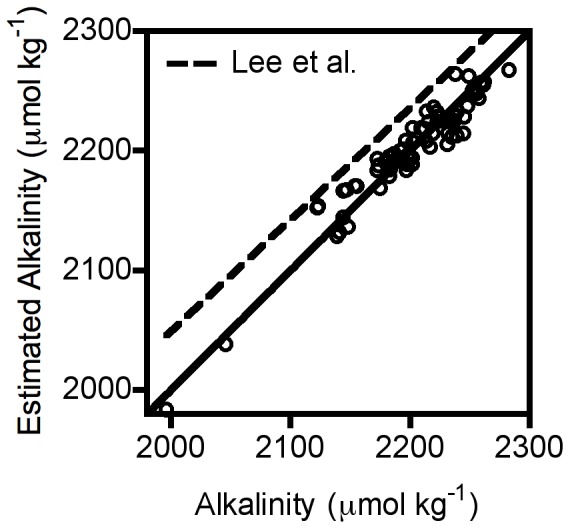
Relationship between measured alkalinity of water sample and estimated alkalinity based on temperature and salinity measurements made by an *in situ* probe as the water sample was collected. Dashed line: best fit line of alkalinity estimates derived from empirical relationship for the North Pacific reported in [24] and used previously in [2]. Open circles: estimated alkalinity based on custom regression between measured alkalinity and measured salinity and temperature for our site. Solid line: measured alkalinity = estimated alkalinity.

The total alkalinity of our samples measured by the CDQC laboratory was linearly related (r = 0.963, Fig. 3) to estimates derived from temperature and salinity based on previously published empirical relationships [24], which we had used in our prior work [2]. These estimates, however, deviated substantially from an identity relationship, generating overestimates of alkalinity, particularly at high true alkalinity (Fig. 3). Therefore, we generated a new custom relationship for our site using stepwise regression with quadratic and linear temperature and salinity terms as in [24]. This procedure supported a direct linear relationship with salinity only (TA = 894.17+40.49 Salinity; r = 0.961; Fig. 3).

**Figure 4:**
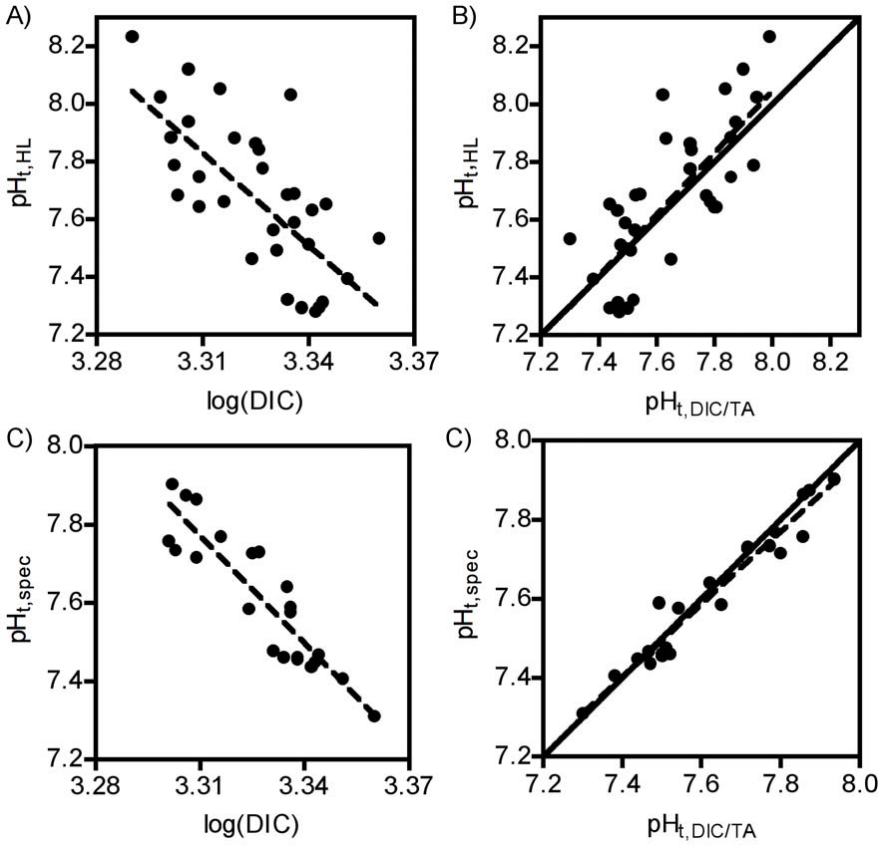
Relationships between pH measurements and DIC-derived measurements. Top row: relationships with pH measured with in-situ probe at time of water collection, Bottom row: relationships with pH of concurrently collected water samples measured spectrophotometrically. Left column: relationship of DIC concentration to pH measures, Right column: relationship to pH calculated from DIC and TA measurements using CO2SYS program. Solid lines: identity relationship, dashed lines: lines of best fit from linear regression.

Dissolved CO_2_ concentrations, in conjunction with TA, could largely explain the variability and low pH observed at our site in recent years. Our in-situ pH measurements (total scale) were negatively associated with log(DIC) values measured by the CDQC laboratory (Fig. 4A, linear regression, pH = 42.18–10.38 log(DIC), r = −0.703), and exhibited a relationship with pH_total_ calculated from measured DIC and TA that did not differ from the identity relationship (Fig. 4B, linear regression, pH_HL = _−0.11+1.018 pH_DIC_, r = 0.756, p(pH_HL_ = pH_DIC_) = 0.908). Spectrophometric pH measurements from the lab showed a tighter association (Fig. 4C, linear regression, pH_spec_ = 38.16–9.18 log(DIC), r = 0.911). These measurements also exhibited a relationship to pH_total_ calculated from DIC and TA that did not differ from the identity relationship (Fig. 4D, linear regression, r = 0.971, p(pH_spec_ = pH_DIC_) = 0.121).

**Figure 5:**
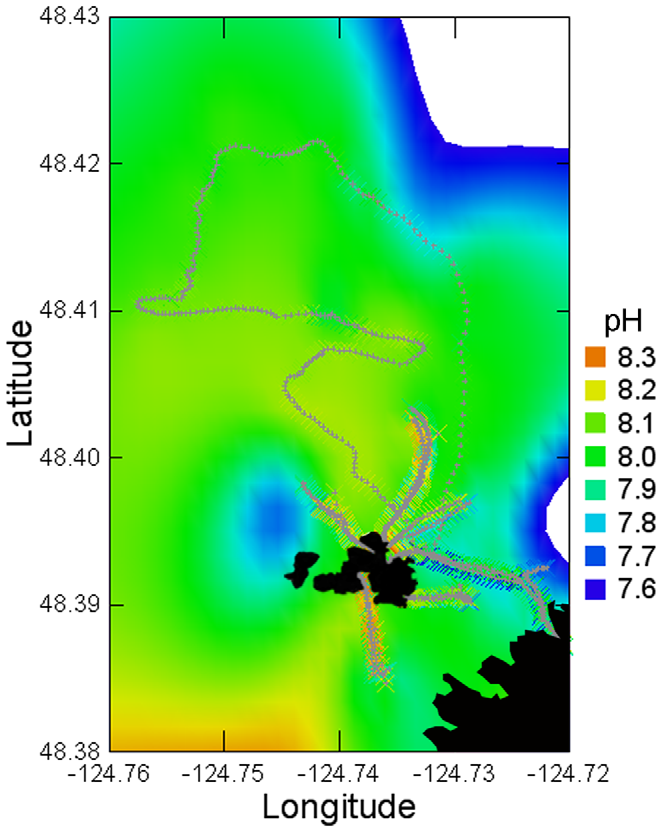
Spatial pattern of pH (NIST scale) measured at 10 sec intervals from boat transects radiating from Tatoosh Island, Washington. Color scale varies from low pH (blue colors) to high pH (red colors). Transect measurement points indicated with small gray crosses superimposed over larger X's color coded for the pH of the sample. Transects spanned depths of 2 m (within 10 m of shore) to 210 m (Juan de Fuca trench in the northwestern area of the plot). Data are presented as a smoothed surface using all transect points and applying distance-weighted least squares with SYSTAT, ignoring the presence of Tatoosh Island and Cape Flattery (black). Note that most rapid color changes occur as extrapolations outside the area of the data, and therefore are unreliable.

Measurements in transects leading away from Tatoosh Island (Fig. 5) showed no evidence that this site exhibited anomalous pH conditions compared to elsewhere in the region. If Tatoosh readings were unusual, pH measurements would exhibit a “bullseye” pattern around the island, but there was no evidence for this pattern in the regional transects that we sampled (Fig. 5, Table 1). Data from other sites around the Strait of Juan de Fuca taken by the Washington State Department of Ecology also generally exhibited much more rapid rates of decline on average (−0.018 yr^−1^) than predicted by global models of pH (Table 2).

**Table 1:** Mean±s.d. pH (NIST scale) from offshore surface samples taken by boat along a 85 km transect into the Strait of Juan de Fuca.

Site	Lat Long	km From Tatoosh	pH_NIST_
Mouth of Strait	48.167–124.732	1.9	7.862±0.054
Second Beach	48.270–124.561	13.0	7.961±0.034
Slip Point	48.382–124.247	38.7	7.964±0.028
Observatory Point	48.409–123.621	86.4	7.937±0.054

Site names represent reference points on the adjacent shore. Samples collected 26 June, 22 July and 26 August 2010 (n = 3 per site-date).

**Table 2:** Trends (Δ) in pH (NIST scale) taken from sampling stations in the inner Strait of Juan de Fuca by the Washington State Department of Ecology.

	Latitude			ΔpH yr^−1^	
Site	Longitude	Date Span	N	(SE)	P(ΔpH <0)
Admiralty Inlet	48.0300	10/26/92–	88	−0.014	0.038
	−122.6167	4/26/04		(0.008)	
Admiralty Inlet North	48.1875	1/9/89–	120	−0.017	<0.001
	−122.8417	11/15/05		(0.005)	
Lopez Sound	48.5133	11/20/90–	191	−0.009	<0.001
	−122.8500	12/12/07		(0.003)	
Strait of Georgia	48.8083	2/13/89–	116	−0.023	<0.001
	−122.9533	11/2/05		(0.007)	
Morse Creek	48.1217	1/9/89–	38	−0.028	0.072
	−123.3500	9/21/94		(0.019)	
*Average*				−*0.018*	*<0.001*

### Trends in Potential Drivers

**Figure 6:**
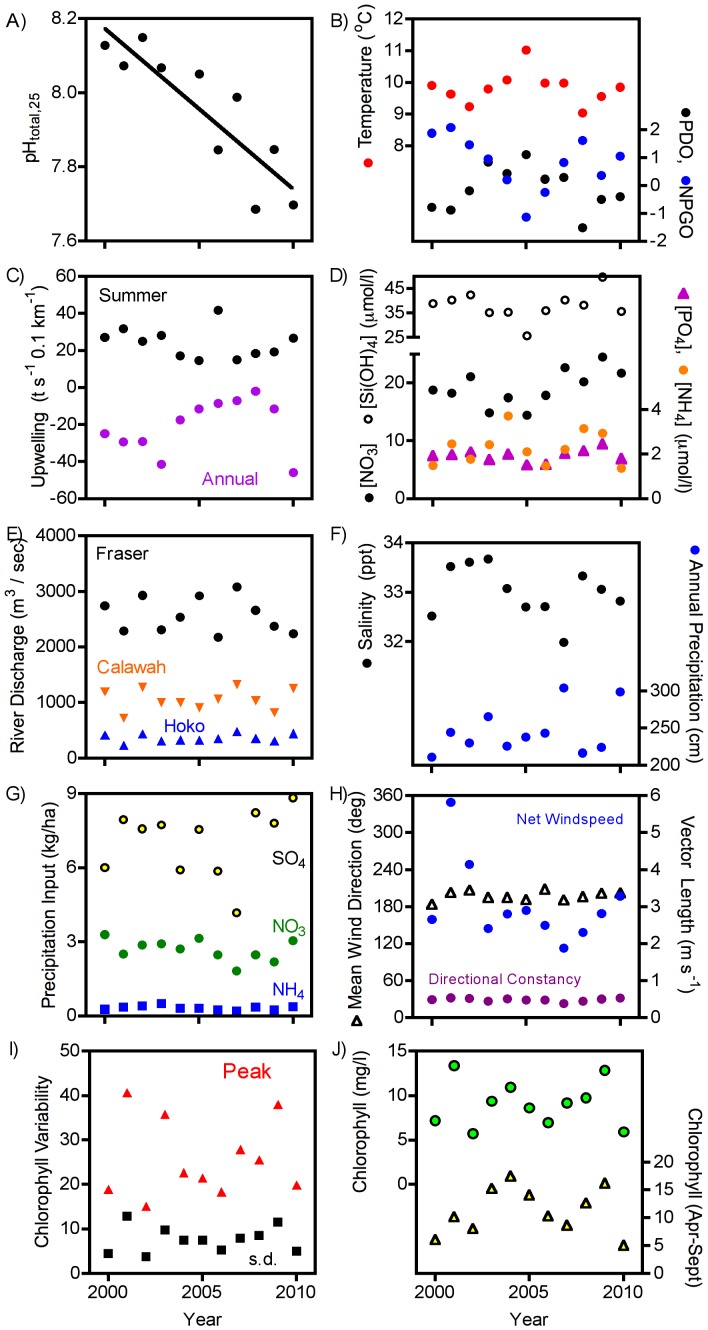
Temporal trends in annual means of pH and potential environmental drivers of pH. A) pH, B) Temperature, PDO and NPGO, C) Upwelling (summer average only, annual average), D) Nutrients (PO_4_, Si(OH)_4_, NO_3_, NH_4_), E) Discharge from three rivers, F) Salinity and precipitation, G) Precipitation chemistry, H) Local winds (mean direction, average wind speed and relative constancy), I) Phytoplankton chlorophyll variability (peak value, standard deviation), J) Average phytoplankton chlorophyll (summer only and average annual). Significant (p<0.05) regressions indicated with curves.

Aside from atmospheric CO_2_ (data not shown), annual means of the variables we probed that should be linked to other key hypothesized drivers of ocean pH generally showed no significant (p<0.05) trends through time, in contrast to the strong trend in ocean pH (Fig. 6A). Average water temperature did not change consistently through time, nor did the PDO or NPGO indices, although these tended to vary inversely with each other and exhibited cyclical patterns over the duration of our measurements (Fig. 6B). The average annual wind-driven upwelling index did not change through time, either when accounting for the entire year or the period when our measurements were taken (Fig. 6C). Dissolved nutrient concentrations exhibited no significant trend through time, although nitrate showed a possible fluctuation that might be associated with temperature and the PDO (Fig. 6D). Freshwater input showed no evidence of directional change through time: annual river discharge at the closest local, largest local, and largest regional river exhibited no temporal trends, nor did salinity or local precipitation (Figs. 6E,F). The chemical composition of rainwater did not vary through time in a direction consistent with reducing pH (Fig. 6G). Local wind characteristics, which might be better indicators of local upwelling patterns, also did not vary in direction or speed (Fig. 6H). Phytoplankton chlorophyll did not change through time either in its monthly variability (Fig. 6I) or in its mean abundance (Fig. 6J). There was also no evidence of increased loading of DOC into the coastal ocean, as DOC values both in local rivers and in large regional rivers tended to decline, rather than increase as predicted (Table 3). Overall, there were no significant (p<0.05) pairwise correlations between pH and any of these variables.

**Table 3:** Trends in DOC concentrations in rivers over time in northwestern North America (Washington state, USA and British Columbia, Canada) over recent decades.

River (Site)	SamplingPeriod	Trend(mg l^−1^ yr^−1^)
*Local Rivers*		
North Fork Sol Duc (Upper)	7/2001–7/2010	−0.052
Main Sol Duc (Upper)	7/2001–7/2010	−0.050
South Fork Sol Duc (Lower)	7/2001–7/2010	−0.932
South Fork Calawah (Upper)	7/2001–7/2010	−0.097
South Fork Calawah (Lower)	7/2001–7/2010	−0.415
North Fork Calawah (Upper)	7/2001–7/2010	−0.164
Sitkum (Lower)	7/2001–7/2010	−0.153
Clallam (Upper)	7/2001–7/2010	−0.055
Clallam (Lower)	7/2001–7/2010	−0.814
South Fork Pysht (Upper)	7/2001–7/2010	−1.198
Main Stem Pysht (Lower)	7/2001–7/2010	−0.372
Little Hoko (Lower)	7/2001–7/2010	−0.117
*Washington Rivers (USGS)*		
Columbia (Richland)	1/1979–4/2010	−0.033
Yakima (Kiona)	1/1978–9/2004	−0.063*
Elwha (Port Angeles)	10/1977–4/1994	−0.107
Columbia (Priest Rapids)	7/1978–4/2010	−0.053*
Big Soos Creek (Auburn)	12/1995–8/2007	−0.175
Thornton Creek (Seattle)	3/1996–9/2007	−0.066
Crab Creek (Ritzville)	12/1992–9/2004	−0.100*
Andrews Creek (Mazama)	2/1981–8/2009	−0.084
Swan Creek (Tacoma)	8/1983–7/2007	−0.061
Naches (Yakima)	4/1987–3/1990	−0.201*
Yakima (Union Gap)	10/1979–8/1999	−0.161*
Granger Drain (Granger)	6/1991–9/2004	−0.242*
*British Columbia Rivers (Environment Canada)*		
Columbia (Birchbank)	11/1997–12/2006	0.102*
Columbia (Nicholson)	4/2003–10/2006	−0.113
Columbia (Waneta)	11/1997–12/2006	0.058*
Fraser (Hope)	11/1997–12/2006	−0.021*
Fraser (Hansard)	12/1997–6/2006	−0.523*
Fraser (Marguerite)	11/1997–12/2006	−0.076*
Fraser (Red Pass)	11/1997–12/2006	0.031*
Meyers Creek	2/1998–9/2004	0.079*
Sumas River	2/1998–12/2006	0.078*

Most rivers show declining trends, in contrast to rivers draining into the North Atlantic. *P<0.05.

Trends in average annual values of environmental parameters were largely the same when analysis was done at higher temporal resolutions (Table 4). Although most parameters continued to show no trends over time, there were three exceptions, all of which had data available at extremely high sample sizes (n >4000): water temperature (30 min sample interval), salinity (30 min interval) and daily upwelling (daily estimate throughout the year). In all of these cases, the amount of variation explained by the temporal trend was very small (1.2% or less). The estimated slopes through time were very slight relative to the typical variation for these variables. Specifically, the upwelling slope was estimated to be 1.5 on an index that varies >1000 units over a year and this slight trend was attributable to changes in the strength of downwelling during winter months. Similarly, the temperature coefficient would, at most, indicate a change of only −0.1°C over the past decade. Based on the empirical relationship of these variables with pH at our site [2], these rates of change are not explanatory for the rapid pH decline we observe (estimated effects on annual pH change: Upwelling: −0.007 per year, Temp: −0.0017 per year, Salinity: 0.012 per year). The trend in the upwelling index is driven by weaker rates of downwelling during November-December, half a year before our measurements were taken. We know of no mechanism that would cause such a delayed response.

**Table 4:** Annual trend statistics for variables at higher-frequency sampling intervals than yearly averages, derived from slopes of linear regression versus year.

	Sampling				
Variable	Interval	n	r	Trend (yr^−1^)	p
WaterTemperature*	30 min	38,997	−0.022	−0.009	<0.001#
Salinity*	30 min	38,997	−0.111	−0.044	<0.001#
Precipitation	Daily	4013	0.010	1.202	0.546
Upwelling	Daily	4015	0.049	1.564	0.002#
Upwelling*	Daily	624	−0.017	−0.220	0.678
NO_3_ deposition	Monthly	122	−0.033	−0.001	0.720
SO_4_ deposition	Monthly	122	0.166	0.007	0.067
NH_4_ deposition	Monthly	122	0.016	0.000	0.859
Precipitation*	Monthly	53	0.092	0.192	0.507
PDO*	Monthly	53	−0.110	−0.032	0.430
NPGO*	Monthly	53	−0.238	−0.072	0.084
NO_3_ *	Monthly	53	0.176	0.391	0.203
NH_3_ *	Monthly	53	0.085	0.041	0.543
PO_4_ *	Monthly	53	0.061	0.010	0.660
Si(OH)_4_ *	Monthly	53	0.092	0.328	0.527

Variables with very high sample sizes have statistically significant trends, but these relationships typically explain at most only a few percentage points of the variability.

* Data from periods coincident with pH data collection only.

# Statistical significance retained after correcting for first-order autocorrelation of time series data.

**Figure 7:**
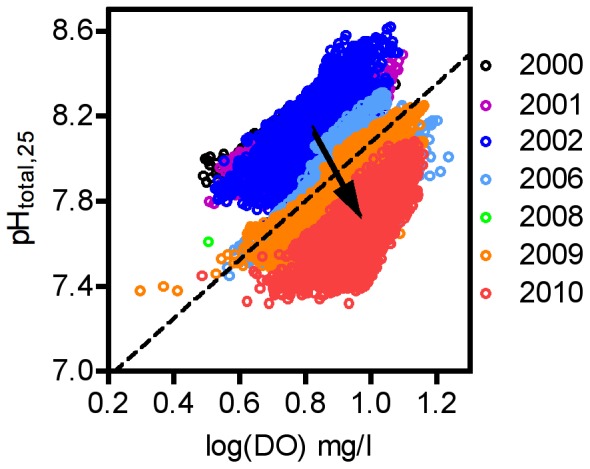
Patterns in the relationship between dissolved oxygen and pH within and among years. Years when oxygen probe (2003, 2005, 2007) or pH probe (2004) malfunctioned are not included. Note that relationships within years (single colors) follow the positive relationship expected between the two from the photosynthesis-respiration relationship of CO_2_ and O_2_ (dashed line), but the trend across years (vector) does not follow this relationship.

Dissolved oxygen is expected to vary with pH because of the role of photosynthesis and respiration in removing or generating CO_2_ in the water. Within years, pH and log(DO) fluctuate positively in tandem with the expected stoichiometry as aerobic respiration takes up O_2_ and generates CO_2_ (lower pH) while photosynthesis does the opposite (Fig. 7). Between-year variation in DO and pH, however, follows a very different pattern: DO tended to increase through time, and hence correlated negatively with pH.

## Discussion

### Overall pH Trends

Our long-term monitoring data show that pH continues to decline in coastal Washington at a rate that is an order of magnitude greater than predicted by models (Fig. 1). While our long-term-studies have focused on a single site, Tatoosh Island, the levels of pH that we observe there are seen at a broader scale. Aside from our spatial data that show no evidence of unusually different pH levels at Tatoosh (Fig. 5) and from parallel trends observed in the eastern Strait of Juan de Fuca (Table 1), observations of high pCO_2_ values from the broader Strait of Juan de Fuca [18] and coastal Oregon [25] are consistent with the low pH levels we have observed in the latter portion of our data, suggesting a regional scale pattern. Furthermore, the high rates of pH decline reported in the North Sea [17] suggest that mechanisms creating these patterns may be operating at a more global scale.

Concerns about measures made with standard electrochemical pH probes have been raised [26], [27] due to their lower accuracy, potential to drift, and effects of varying ionic concentrations of salt water on their performance. Under some circumstances, these features could lead to bias in observed rates of change, possibly leading to an over-estimation of rates of pH change. The correspondence of our electrochemical measures with other more accurate measures suggests that our results are not systematically biased. Our short intervals between probe maintenance relative to the time scales of the patterns being investigated, and statistical procedures that explicitly extract potential drift may help in this case. Although higher error in electrochemical methods could compromise the ability to detect a statistical pattern, this does not seem to be an issue in our data set, as temporal trends are readily apparent despite this error (Fig. 1) and the large majority of the variation in our pH measures is explained statistically by a limited set of relevant variables [2].

### Testing Possible Drivers

Given the evidence that our measurements reflect the general pH conditions of the area, we are then left with a more perplexing question: Why is pH declining so rapidly in this area compared to global model predictions assuming equilibrium conditions with the atmosphere? Our analyses of DIC and TA are in general agreement with those generated by our spectrophotometric and electrochemical measurements of pH, indicating that the low instrumental pH values are consistent with other measurements of the inorganic carbon content of the water.

Increasing delivery of low pH water from below the surface is an appealing, straightforward hypothesis to explain the declines (hypothesis #1), but examination of multiple lines of evidence provides no clear independent support. There is little doubt that upwelling of subsurface waters with high concentrations of DIC derived from respiration contributes to low pH conditions at some times of the year. Observations in 2007 at transects radiating from the west coast of North America reveal low pH waters lurking close to the surface in near-shore areas characterized by upwelling [19]. Additionally, we observe a strong contribution of upwelling to pH variability [2]. These observations at one point in time do not mean, however, that low-pH, upwelled water drives the observed overall temporal decline. Although we explored 7 different variables expected to be associated with upwelling, none of these showed consistent, quantitatively significant trends through time that would support the increased upwelling hypothesis (Fig. 6). Relevant variables included the widely-used regional upwelling index based on surface winds, local surface wind patterns, cooler water temperatures, higher nutrient concentrations, or lower oxygen concentrations. Additionally, shells of the California mussel (*Mytilus californianus*) from our study site exhibit a rapid decline in their δ^13^C values (−0.071°/_oo_ per year) coincident with our measured pH decline but exhibit no temporal patterns in δ^18^O expected with upwelling associated cold temperature waters [28].

Although changes in eutrophication (hypothesis #2) have been hypothesized to drive changes in coastal pH in other areas [17], our data do not provide support for this hypothesis. Typically, increased eutrophication is expected when more anthropogenic nutrients are introduced and become available for primary production. None of the major nutrients we routinely measured in the water exhibit a systematic increase through time, so there appears to be no additional capacity to stimulate eutrophication in the area. Furthermore, there is no evidence of increased overall phytoplankton abundance nor the magnitude of phytoplankton blooms over the course of our measurements, either in satellite-based chlorophyll estimates (Fig. 5g–h) or in long-term water-sample data from the top 20 m at Fisheries and Ocean Sciences Canada Station P (50°N 145°W) located 1500 km west-northwest of our site (2000–2009, coefficient = −0.029, p = 0.062, based on data from [21]). These latter data also do not strongly support the hypothesis (#6) that changes in the phytoplankton are affecting pH through reductions in carbon uptake from the surface waters or increases in organic carbon export to the subsurface water and subsequent increases in respiration-generated CO_2_ concentrations that return via upwelling.

Reduced buffering capacity via increased freshwater input (hypothesis #3) is another appealing hypothesis that fails to receive any independent support in the data series we have explored. Global warming from anthropogenic input of CO_2_ into the atmosphere is predicted to increase precipitation in many areas, including northwestern North America and rising global air temperatures could also increase glacial melting from local mountains [29]. As products of global warming, these mechanisms could act in concert with diffusion of CO_2_ into the ocean to generate a strong pH-CO_2_ relationship. These hypotheses predict that precipitation should increase, river discharge should increase and, importantly, that these increases create an appreciable effect on seawater concentrations of solutes. None of these variables, however, exhibit long-term trends over the course of our pH data series.

A variant of the eutrophication hypothesis (hypothesis #4) would be that dissolved organic carbon is being introduced into the coastal ocean at increasing rates, increasing respiration-generated CO_2_. Rivers draining into the North Atlantic have shown a general increase in DOC concentrations [22], [30], suggesting a general increase in oceanic organic carbon loading is occurring. One explanation for these patterns is that higher CO_2_ levels and associated terrestrial plant production lead to greater input of organic carbon into rivers [31]. This potential link with CO_2_ is intriguing, as it would reinforce the strong association observed between our observed pH change and atmospheric CO_2_. Alternative explanations exist, however, including the effects of declining acid precipitation on soil chemistry [22]. Available data for Washington and British Columbia show a different pattern, with DOC concentrations tending to decline in rivers. A similar pattern is seen in the Yukon River basin [32]. These patterns therefore fail to support the direct organic carbon input hypothesis for declining ocean pH, and are consistent with the hypothesis [22] that declining acid precipitation is a better explanation for observed increases in the North Atlantic basin. Whereas higher atmospheric CO_2_ concentrations are a global-scale pattern, acid precipitation is a more regional phenomenon that was historically a minor issue along the northeastern Pacific coast, owing to the lack of upwind industrial activity.

Although not historically a factor, increasing industrialization of southeast Asia raises the possibility that acid precipitation input is increasing across the Pacific, which might lead to systematic declines in pH (hypothesis #5). Calculations indicate that this mechanism is not sufficient to generate significant ocean acidification on a global scale, but we address it for completeness here. Data from the nearby station monitoring rainfall chemistry does not support this hypothesis, as concentrations of the major constituents causing acid rain showed no significant trends through time and, if anything, declined. Acid precipitation falling in other areas of the ocean conceivably could be transported via water without being present in local rainfall. The lack of change in NO_3_ and NO_2_ concentrations in our water samples are inconsistent with this variant of the hypothesis, but we know of no SO_4_ data to more fully test it.

The introduction of organic carbon and its subsequent respiration to generate CO_2_ is a central component of several hypotheses to explain rapid pH decline. Because respiration generates CO_2_ through the use of O_2_, there should be a tight positive link between DO and pH. Although localized hypoxia events have been reported in central Oregon [33] and near the Columbia River [34], we have detected no such events in association with declining pH at our site. Respiration-generated pH changes would result in a relationship between pH and DO among years that slides down the stoichiometrically-appropriate slope as pH declines over the span of our data. The observed pattern in our data does not support this hypothesis: although the expected tight positive link between O_2_ concentration and pH exists within years, it does not appear among years. The DO data therefore are at odds with the upwelling hypothesis (low pH generated by organic decomposition in the subsurface waters), the eutrophication hypothesis (high nutrients generate increased primary productivity, which then decomposes), or the increased DOC input hypothesis (increased terrestrially-derived organic carbon enters the ocean, where it decomposes).

Total alkalinity at our site (Fig. 3) tends to be lower than expected under similar salinity and temperature conditions for the broader North Pacific [24]. The causes for this discrepancy remain to be identified, but the reduced alkalinity could create conditions more conducive to larger pH declines through lowered buffering of the seawater. Salinity at our site (33 ppt) is low relative to many areas of the world, but is typical of the North Pacific [35], from which the relationship of Lee et al. [24] was derived. Because salinity is tightly associated with TA, but shows no change through time, however, changes in alkalinity per se seem unlikely to explain the declines in pH we have observed. Nonetheless, the causes and implications of the lower alkalinity at this site deserve further attention, particularly whether parameters in the salinity-TA relationship become time-dependent over a longer-study duration.

In sum, our tests of multiple hypotheses for the causes of the rapid pH decline in this region of the California Current have not revealed a definitive driver. Despite our extensive tests of multiple datasets for expected potential drivers, we have been unable to uncover a “smoking gun” of systematically changing environmental parameters through time that are known to be relevant to ocean pH, with the exception of atmospheric CO_2_ concentrations. These pH changes have been accompanied by a rapid decline in the δ^13^C (but no change to δ^18^O) in mollusk shells that is historically unprecedented over the last 1000 years at Tatoosh Island [30]. The isotopic changes are 2.5× faster than expected by the Seuss Effect, have no associated stoichiometric changes to nitrogen, and suggest increased input of biologically-processed carbon, either from present-day sources or fossil sources. The rate of change in shell δ^13^C at Tatoosh is also comparable to that reported for coralline algae in the Gulf of Alaska [36]. Given the observations of rapid pH declines in areas of the North Sea [18, L. Peperzak pers. comm.], we suspect that causes of this pattern may be not be restricted to the Washington coast.

Rates of pH decline that are more consistent with model predictions have been observed in series generated in Hawaii [3], Bermuda [4] and the Canary Islands [5]. Comparison of these areas to sites exhibiting more rapid declines may therefore offer clues for further study. Areas with known rapid rates of pH decline are coastal, cold water, and high-productivity sites where there are strong feedbacks between the biota and seawater chemistry. Ocean pH predictions are based on assumptions of chemical equilibrium between oceans and the atmosphere, whereas our data show that pH is highly dynamic due to daily and seasonal fluctuations in photosynthesis, upwelling and water temperature [2]. Non-linear dynamic theory developed for other areas of ecology shows that average behavior of non-equilibrial systems can vary markedly from predictions based on behavior of equilibrium points, including magnifying responses to external inputs [37], [38]. Perhaps similar non-equilibrial effects apply to the non-linear inorganic carbon system if atmospheric CO_2_ input into the water column during periods of low DIC in the water occurs more rapidly than release of CO_2_ when water concentrations of DIC are high. More multifaceted scenarios involving spatio-temporal variation in processes driving pH decline in conjunction with differential flux rates into/out of the water and complex coastal currents might also be plausible, if they can explain the decoupling of DO and nutrients from respiration-derived CO_2_ that would be consistent with our data. For example, O_2_ has higher flux rates than CO_2_ into and out of solution [39]. A more extensive regional network of spatial and temporal sampling would be necessary to identify up-current hotspots for all three parameters in conjunction with a gradient of moderating effects of nutrient and DO concentrations in the direction of our site. In any event, given the failure of multiple hypotheses to explain rapid rates of pH decline in our study, the importance of coastal ecosystems to fisheries, and evidence that key species such as oysters and mussels are being impacted by declining pH [2], [40], [41] and carbonate ion abundance [42], further research into dynamics of the coastal carbon cycle is warranted.

## Study Site, Methods and Data Sources

### Ethics Statement

All necessary permits were obtained for the described field studies. Specifically, all research was conducted from Makah tribal lands, for which we received the written permission required for access to Tatoosh Island.

### Core Water Sampling Data

Focal data sets were collected from Tatoosh Island, Washington state, USA (48.32°N, 124.74°W), located in the northeastern Pacific at the mouth of the Strait of Juan de Fuca, 0.7 km from the mainland of the Olympic Peninsula. Tatoosh has been a site of intensive ecological investigation since 1969 [43], [44]. Beginning in 2000 we have collected water data from late spring through late summer (generally late April-early September) at half hour intervals using Hydrolab DataSonde 4a/5× data loggers which included probes for electrochemically-measured pH, water temperature, dissolved oxygen, and salinity [2], [24]. During visits to the site, usually every two weeks, units were serviced, which included downloading data and calibrating probes (NIST pH standards of 7 and 10, saturated air O_2_ calibration), and then replacing the unit 12–24 hr later. The unit was bolted to the vertical wall of a 5000 l tide pool that is separated from the ocean during the lowest tides (80 cm MLLW) by a 1 m wide wall of rock with a 5 m vertical drop-off into the ocean. Contemporaneous measurements made in the pool and adjacent ocean with handheld probes showed that the two do not differ in temperature, salinity, pH, and DO (all r^2^>0.95) [2]. In our analyses, we exclude any data taken when predicted tide height is <80 cm to further insure that measured conditions are those of the near shore ocean, rather than an isolated tide pool. To measure nutrient conditions, we collected 50 ml water samples at monthly intervals (April-September) from 10 stations on Tatoosh Island and four stations along an offshore transect, filtered with a syringe filter through GF/F paper, placed these in acid-washed vials and froze them prior to analysis. The samples were analyzed at the University of Washington Ocean Chemistry Laboratory for PO_4_, Si(OH)_4_, NO_3_^2−^, NO_2_^−^, and NH_4_^+^, using standardized methods [45], [46].

Concerns have been raised about the use of electrochemical probes to measure pH in the ocean [26], [27], as their expected accuracy is an order of magnitude lower than spectrophotometric dye methods, and their performance may be affected by the high ionic content of seawater, potentially causing their readings to drift or be biased over time. Often overlooked in these considerations is that high statistical accuracy can be achieved not only by high measurement accuracy, but also by large numbers of measurements. Hence, the high sample sizes that can be collected by an automated electrochemical system can compensate to some extent for reduced measurement accuracy: our present pH sample size is 50,814 (37,038 after excluding observations when the tide was <80 cm) over the past 11 years whereas a comparable water sampling/wet chemistry program might typically be around 100 for a single station. To control for any systematic sensor drift, we developed a relationship between pH and time since servicing, assumed that the relationship arose from sensor drift, and subtracted the relationship from the data.

To further explore reliability of our measurements, in 2009 and 2010 we collected water samples for lab analysis coincident with times when the probes were sampling the water. First, we analyzed spectrophotometric pH to determine the consistency of our measures, following the general methods of [47]. Sampling occurred opportunistically throughout each field season (n = 13 in 2009, 76 in 2010), when the sampling site was both accessible and connected to the ocean, and represented a range of points in the season (April–August), times of day (0500–2000 h PDT), and tidal current conditions (ebbing/flowing). Samples were collected with a hand-operated 750 ml Van Dorn bottle triggered next to the probes (1–2 m depth), decanted into 10 ml vials which were sealed without air bubbles, stored in a cooler until return from the field (4–120 hr) and incubated for 30 min in a 25°C water bath. A 3 ml subsample was analyzed on a Bausch & Lomb Spectronics 20 spectrophometer with a 1 cm cell width at wavelengths of 434, 578 and 730 nm, before and after additions of one and two 0.2 ml units of 2 µmol/l c-Cresol Purple (Spectrum Chemicals) indicator dye. We followed the formulas in [47] to calculate pH on the total scale. To check the accuracy of our spectrophotometric measurements, we repeated analyses with a subset of samples, and also analyzed pH from a Tris-buffered synthetic seawater standard obtained from the Carbon Dioxide Quality Control Lab at Scripps Institute of Oceanography (Batch #2, pH_total = _8.0928 mol/kg, Salinity = 35 at 25°C). We used linear regression to test for deviations in a relationship of the form pH_2 = _pH_1_, where pH_x_ represent the x-th measurement of each sample. We also used linear regression to test the strength of correspondence between our spectrophotometric and electrochemical pH measures. To check for possible effects of microbial activity on measured pH, we tested for trends in differences in spectrophotometric- and probe-based measures of pH with time since collection, and found none. We also compared pH measurements of an unfiltered versus a filtered subsample (GF/F glass fiber filter paper) from the same cast on 5 different occasions and found no differences. Second, we collected 500 ml water samples (20 in 2009, 16 in 2010), added 120 µl of HgCl to arrest biological activity, and shipped the samples for analysis of salinity, total alkalinity, and pCO_2_ at the Carbon Dioxide Quality Control (CDQC) Laboratory at the Scripps Institute of Oceanography, following the methods of [47]. We regressed our pH probe measurement against the CDQC pCO_2_ measurement to determine the extent to which DIC was associated with variability in pH. We also used the CO2SYS program for Microsoft Excel [48] to calculate expected pH on the total scale at 25°C from pCO_2_, total alkalinity, salinity, and monthly estimates of nutrient concentrations and compared these predictions to those generated by the our estimates of spectrophotometric pH and the pH probe using linear regression testing the hypothesis observed = predicted (pH_total,25_). We converted our electrochemical measurements (calibrated with NIST standards) to the pH total scale to make all pH measurements comparable. To do this, we calculated estimates of both pH_total_ and pH_NIST_ from our DIC/TA data with the CO2SYS program, generated the relationship between these estimates with linear regression (pH_total_ = −0.128+0.999*pH_NIST_, r = 1.000), and used this relationship to convert all our electrochemical pH measurements to the total scale.

### Broad-Scale Water Sampling

The pH trends we observed at our focal sampling site [2] might only reflect very localized changes. We used three sources of data to extend our spatial inferences to a broader scale. First, we carried out several offshore transects to probe for systematic changes in pH with distance from shore. We strapped a Hydrolab Minisonde with pH, DO, temperature, and salinity to the side of a Zodiac boat, so that it was deployed 0.5 m below the surface, and sampled 6 transects radiating in all directions from Tatoosh Island. Data were taken every 10 seconds, travelling at a speed of approximately 8 km/hr, sample locations were recorded with GPS, and water depth was estimated with a sonar depth meter. Transects spanned 0.5–1.1 km in length, and water depths ranging from 2 to 70 m. We also recorded data for 2.25 hr over a longer, more circuitous transect 11 km in length, that extended over the Juan de Fuca Trench (up to 210 m depth and 3.4 km from Tatoosh). Overall, this sampling spanned 8 km^2^ in area. Second, during three dates in 2010 (26 June, 22 July and 26 August) we sampled water offshore aboard the UNOLS vessel R/V Clifford Barnes at four stations along an 85 km transect in the western Strait of Juan de Fuca for pH using a SeaBird SBE-18 sensor calibrated with NIST standards. Third, we analyzed temporal trends from the surface waters (≤2 m) of the inner Strait of Juan de Fuca taken periodically by the Washington Department of Ecology (http://www.ecy.wa.gov/apps/eap/marinewq/mwdataset.asp) using a SeaBird SBE-18 sensor lowered from a float plane. We analyzed the open-water sampling stations in the data set that were sampled for pH over a span of at least 10 years. These were located 105–185 km from Tatoosh over water: Admiralty Inlet, Admiralty Inlet North, Lopez Sound, Strait of Georgia, and Strait of Juan de Fuca near Port Angeles.

### Data Sources for Potential Driver Variables

To address alternative hypotheses of environmental drivers of long-term trends in pH we compiled data from a variety of web-based data sources. To assess upwelling hypotheses, we used monthly wind-derived upwelling indices (http://www.pfeg.noaa.gov/products/las.html) for the closest latitude (48°N, 125°W). To look for potential changes in wind-driven upwelling at the local scale, we also obtained wind information from the NOAA weather station located on Tatoosh Island (http://www.ndbc.noaa.gov/station_page.php?station=ttiw1). We also analyzed two oceanographic indices thought to be associated with changes in currents: the Pacific Decadal Oscillation index (http://jisao.washington.edu/pdo/) and the North Pacific Gyre index (http://www.o3d.org/npgo/data/NPGO.txt).

To address alternative hypotheses relevant to freshwater input and composition, we assembled data from both web-based sources and our own studies of local rivers and precipitation. We obtained monthly precipitation data from the National Weather Service (http://www.ncdc.noaa.gov/oa/climate/climatedata.html) from the closest station available, Quillayute (47°56′N, 124°33′W), and annual river discharge data from the closest (Hoko River, Washington), the largest local (Quillayute River, Washington), and the largest regional (Fraser River at Hope, British Columbia) gauged rivers from either the United States Geological Survey (USGS; http://waterdata.usgs.gov/wa/nwis/rt), or Environment Canada (http://www.wsc.ec.gc.ca/applications/H2O/HydromatD-eng.cfm). We obtained data on dissolved organic carbon (DOC) input from two sources. First, we collected water samples from two sites at each of 9 rivers on the northwestern Olympic Peninsula, Washington, in July 2001 and 2010 for DOC analysis. The sampled rivers flowed through a range of land use levels from relatively pristine (Olympic National Park), to intensively harvested for timber, and included the following: North Fork Sol Duc, South Fork Sol Duc, Main Stem Sol Duc, North Fork Calawah, South Fork Calawah, Sitkum, Little Hoko, Clallam, and South Fork Pysht. Samples were frozen until analysis, which was done by the University of Washington Ocean Chemistry Laboratory following the procedures in [46]. Second, we obtained DOC data from rivers flowing into the larger region of Washington state and southern British Columbia from Environment Canada (http://waterquality.ec.gc.ca/waterqualityweb/) and the USGS (http://nwis.waterdata.usgs.gov/wa/nwis/qw). To assess potential changes in rain chemistry that might be driving pH trends, we analyzed trends in the long-term precipitation chemistry data (http://nadp.sws.uiuc.edu/nadpdata/annualReq.asp?site=WA14) collected from the Hoh River valley (47°86′N, −123°93W), focusing in particular on nitrate and sulfate concentrations that are key components of acid precipitation [49].

We assessed potential effects of changing regional phytoplankton abundance by evaluating satellite-based data of chlorophyll concentration. We obtained monthly data from NOAA for the AquaModis platform (http://coastwatch.pfel.noaa.gov/coastwatch/CWBrowser.jsp) reported at a pixel scale of 0.125 degrees latitude/longitude, and averaged data from all pixels within 48.365° to 49.445°N and 124.725° to 124.795°W. AquaModis data were not available prior to June 2002; for this period, we used data from the SeaWiFS platform, after checking to insure that no abrupt breaks in mean or trend were present when data series were switched. We analyzed four aspects of the data series: annual mean chlorophyll concentration, mean concentration during the deployment season of our probe (April-September), the standard deviation in monthly chlorophyll concentration, and the peak monthly chlorophyll concentration.

### Statistical Analysis

We tested for potential drivers of the observed long-term trend in pH by applying linear regression to different variables through time. Different data are available at different temporal resolutions, ranging from our local measures made at 0.5 hr intervals through spring-summer to annual means. Because statistical power to detect trends can vary with sample size, we carried out analysis on annual means to ensure all data series had comparable power, but also report temporal trends of data series at their highest available sampling resolution for completeness.
